# Supplementary figures and images for: Molecular characterization of Campylobacter spp. recovered from beef, chicken, lamb and pork products at retail in Australia
Source: PLoS One. 2020 Jul 30;15(7):e0236889. doi: 10.1371/journal.pone.0236889 (PMC7392323; doi:10.1371/journal.pone.0236889)

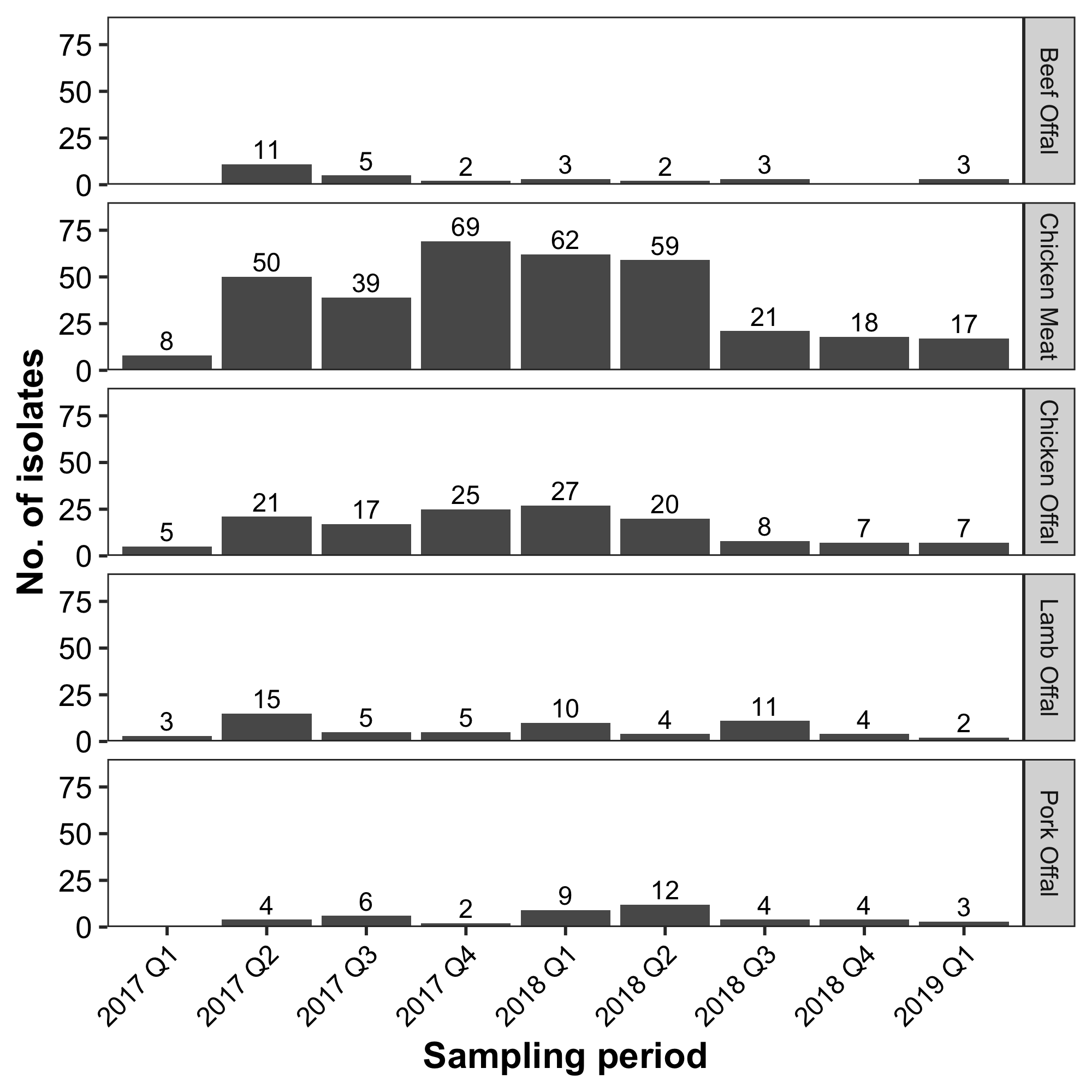

Supplement: S1 Fig — (TIFF) [file pone.0236889.s001.tiff]

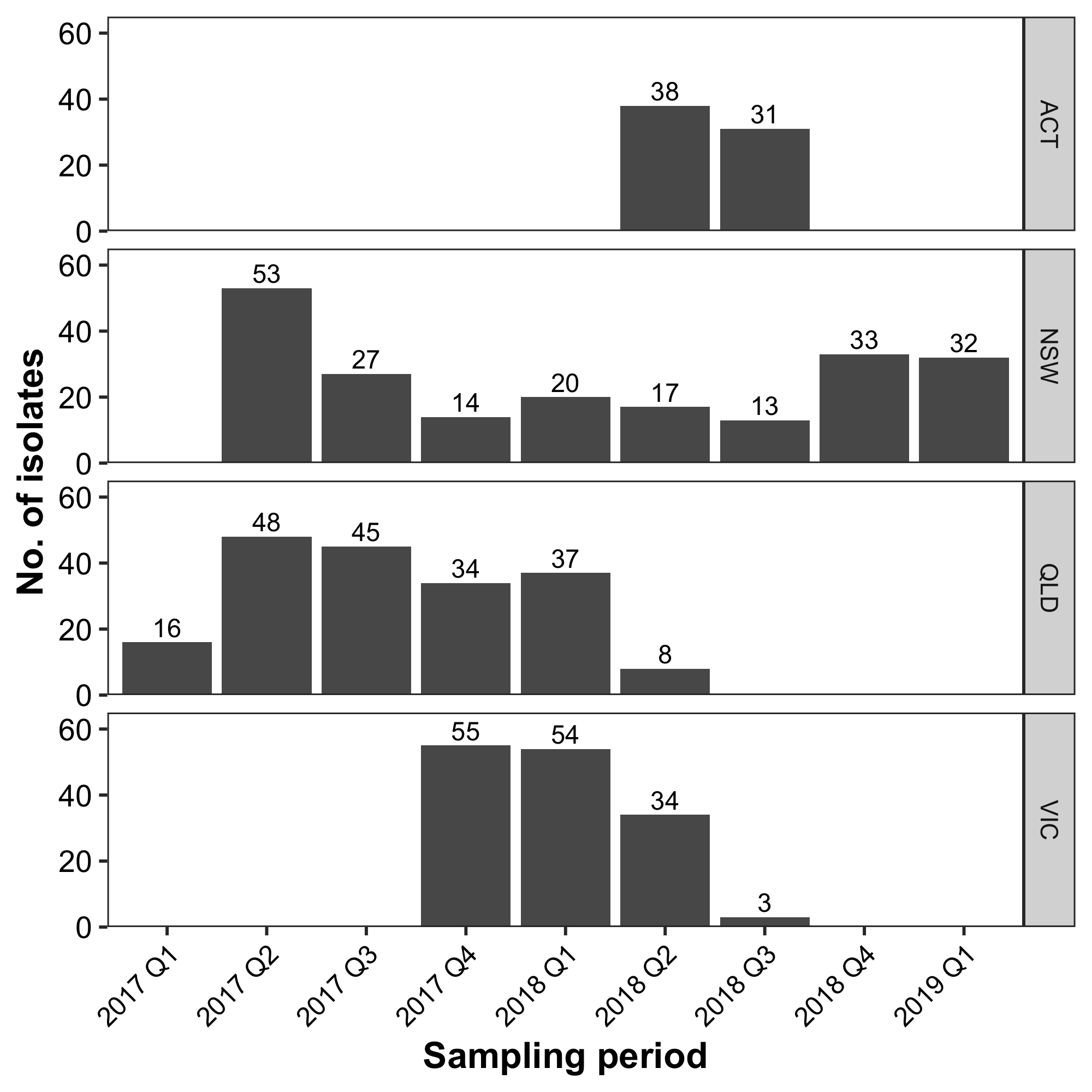

Supplement: S2 Fig — (TIFF) [file pone.0236889.s002.tiff]

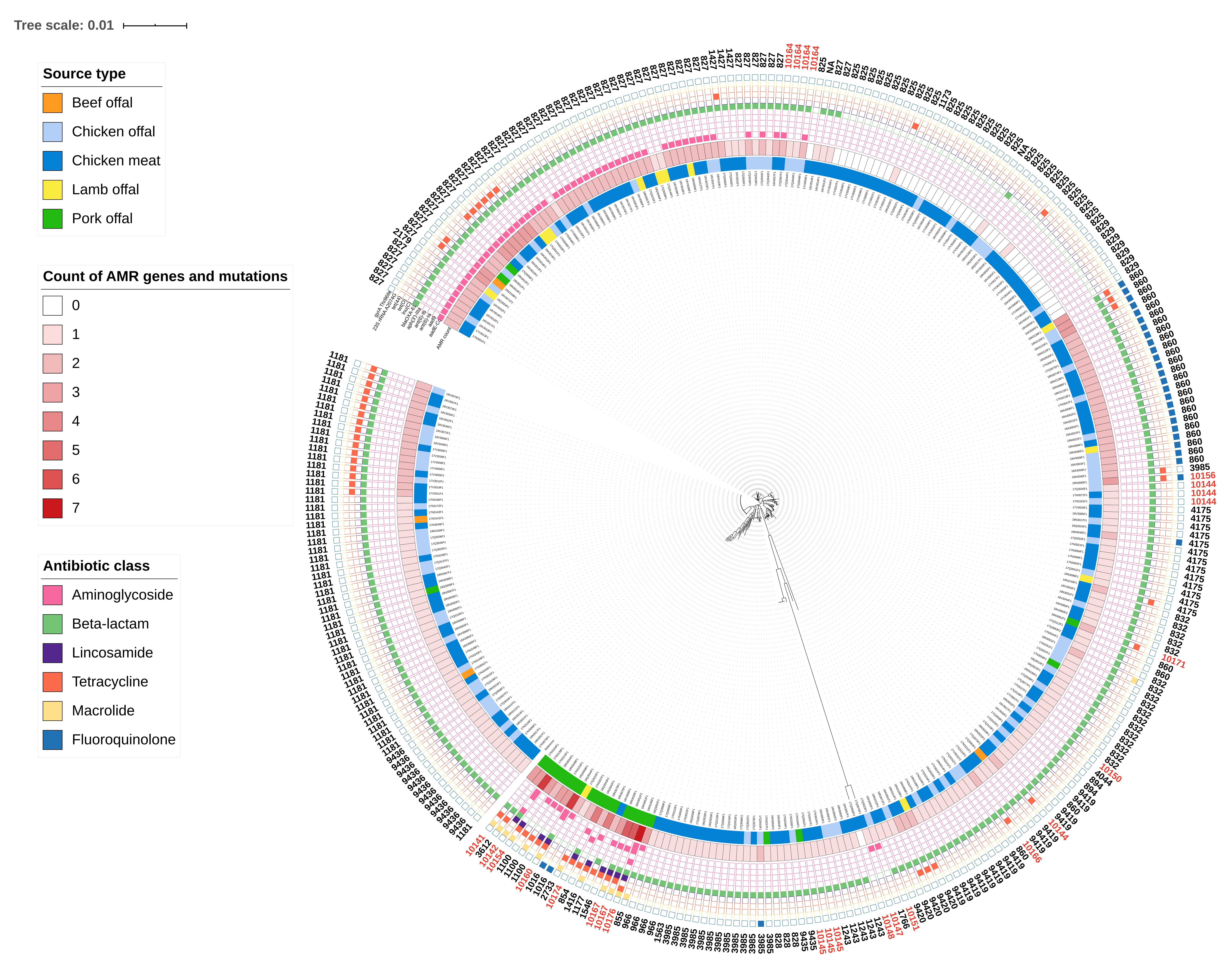

Supplement: S3 Fig — The circle lanes from inner to outer order indicate: food source, number of antimicrobial resistance (AMR) genes or mutations, type of AMR gene or mutation (coloured by antibiotic class) and MLST number. Novel STs identified in this study are shown in red text. (JPG) [file pone.0236889.s003.jpg]

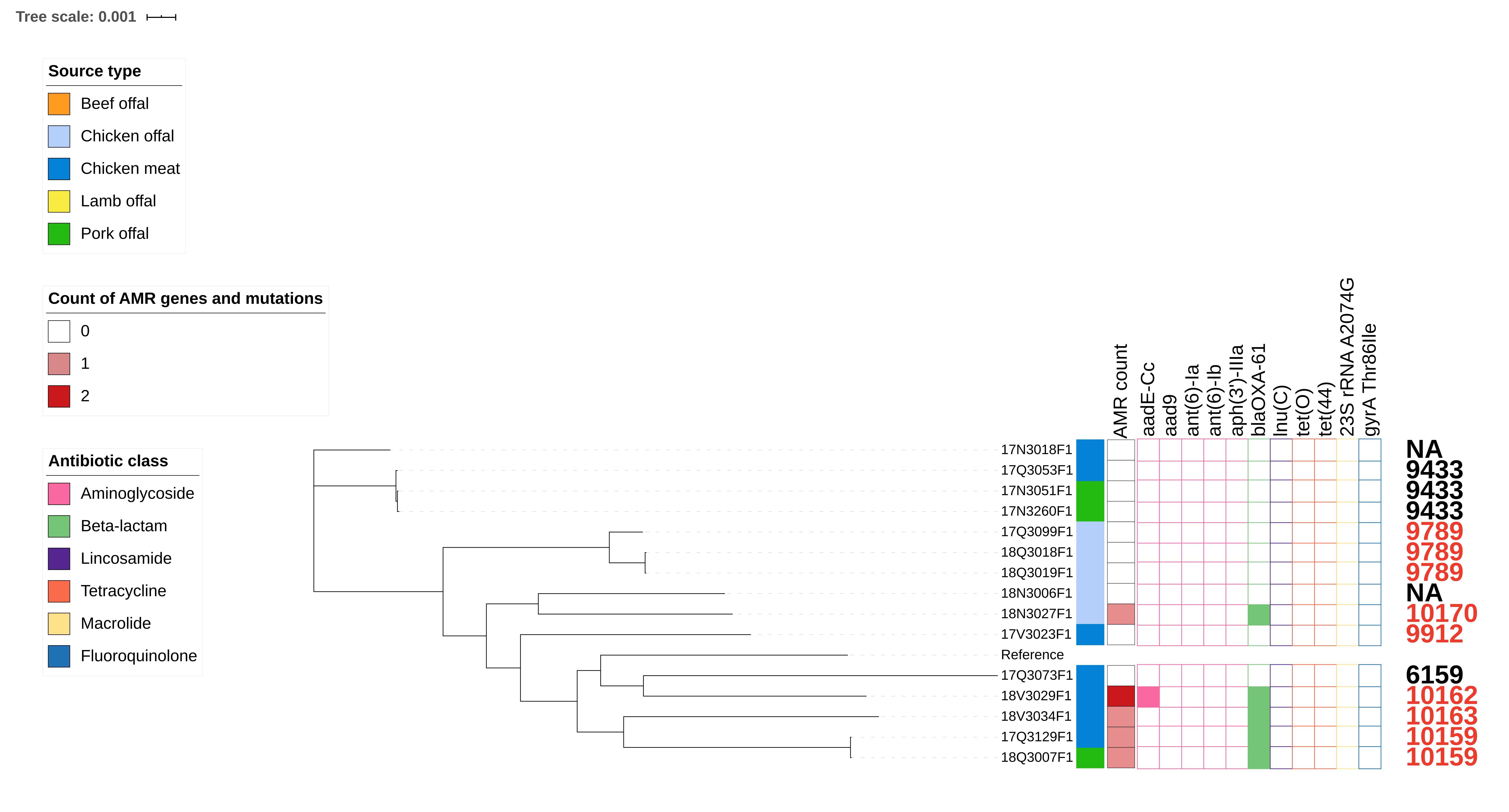

Supplement: S4 Fig — The lanes from inner to outer order indicate: food source, number of antimicrobial resistance (AMR) genes, type of AMR gene (coloured by antibiotic class) and MLST number. Novel STs identified in this study are shown in red text. (JPG) [file pone.0236889.s004.jpg]

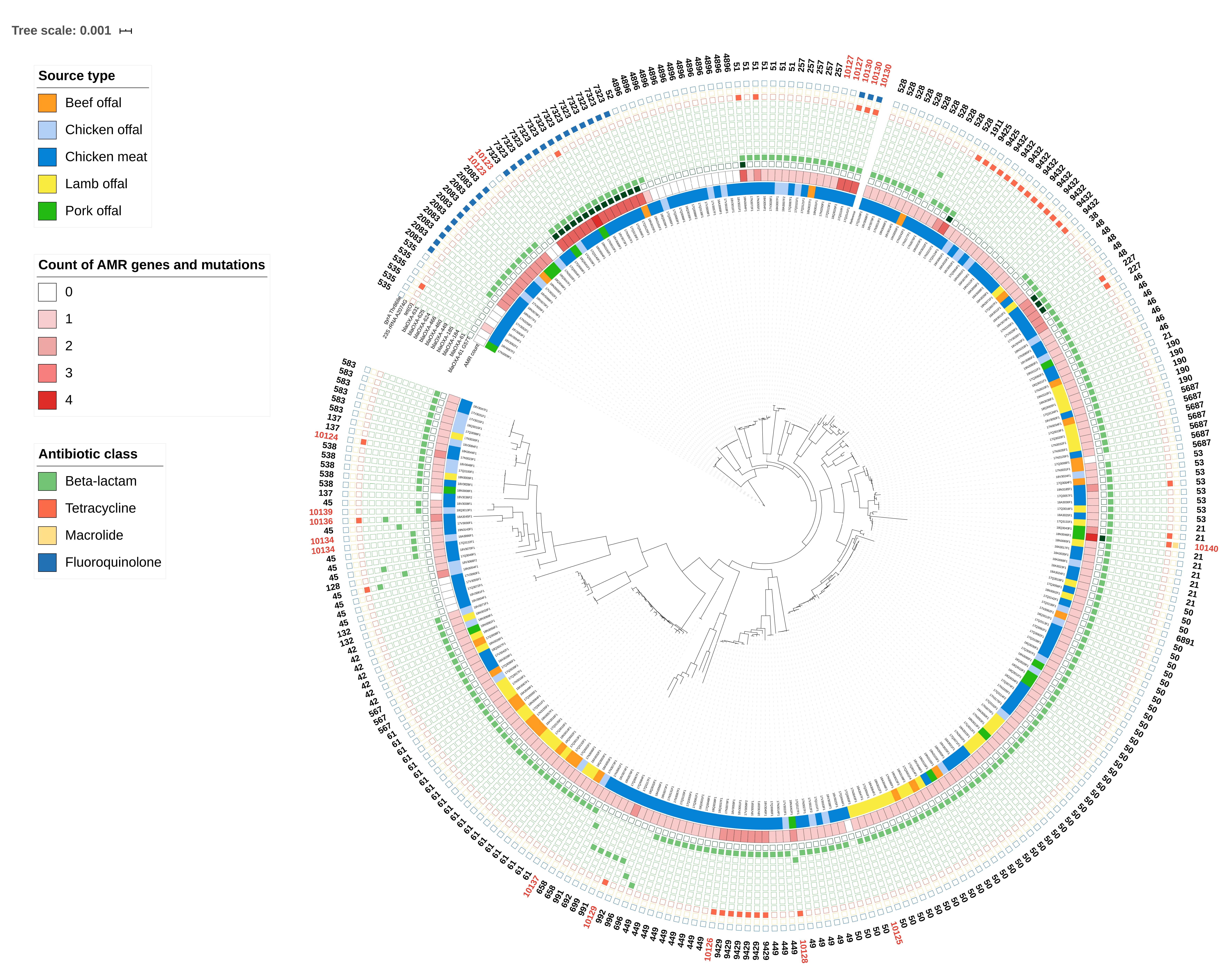

Supplement: S5 Fig — The circle lanes from inner to outer order indicate: food source, number of antimicrobial resistance (AMR) genes or mutations, type of AMR gene or mutation (coloured by antibiotic class) and MLST number. Novel STs identified in this study are shown in red text. (JPG) [file pone.0236889.s005.jpg]
