# Supplementary material for: Molecular characterization of Campylobacter spp. recovered from beef, chicken, lamb and pork products at retail in Australia
Source: PLoS One. 2020 Jul 30;15(7):e0236889. doi: 10.1371/journal.pone.0236889 (PMC7392323; doi:10.1371/journal.pone.0236889)
Supplement: S1 Table — (PDF) [file pone.0236889.s006.pdf]

Table S1

Accession numbers of *Campylobacter coli* and *Campylobacter jejuni* collected from retail beef, chicken, lamb and pork products from Australian retail outlets.

Isolate No.	Species	ST	Source	Accession number
17N3033F1	*C. jejuni*	21	Beef	SRR10537798
17Q3133F1	*C. jejuni*	21	Lamb	SRR10538057
18A3017F1	*C. jejuni*	21	Chicken	SRR10537645
18A3023F1	*C. jejuni*	21	Chicken	SRR10537567
18A3024F1	*C. jejuni*	21	Chicken	SRR10537556
18A3035F1	*C. jejuni*	21	Chicken	SRR10537468
18A3068F1	*C. jejuni*	21	Chicken	SRR10537823
18N3065F1	*C. jejuni*	21	Lamb	SRR10537670
18Q3043F1	*C. jejuni*	21	Pork	SRR10538002
17Q3007F1	*C. jejuni*	38	Beef	SRR10537582
17Q3008F1	*C. jejuni*	42	Beef	SRR10537581
18N3026F1	*C. jejuni*	42	Lamb	SRR10537698
18N3033F1	*C. jejuni*	42	Lamb	SRR10537691
18N3056F1	*C. jejuni*	42	Lamb	SRR10537676
18N3069F1	*C. jejuni*	42	Chicken	SRR10537665
18N3081F1	*C. jejuni*	42	Pork	SRR10537652
18V3071F1	*C. jejuni*	42	Chicken	SRR10537855
17Q3049F1	*C. jejuni*	45	Chicken	SRR10537536
17Q3072F1	*C. jejuni*	45	Chicken	SRR10537511
17Q3122F1	*C. jejuni*	45	Chicken	SRR10538067
17V3030F1	*C. jejuni*	45	Chicken	SRR10537967
17V3055F1	*C. jejuni*	45	Chicken	SRR10537943
17V3060F1	*C. jejuni*	45	Chicken	SRR10537935
18N3054F1	*C. jejuni*	45	Chicken	SRR10537680
18V3008F1	*C. jejuni*	45	Chicken	SRR10537926
18V3070F1	*C. jejuni*	45	Chicken	SRR10537856
17N3065F1	*C. jejuni*	46	Chicken	SRR10537782
17Q3060F1	*C. jejuni*	46	Chicken	SRR10537525
18N3153F1	*C. jejuni*	46	Chicken	SRR10537627
18Q3021F1	*C. jejuni*	46	Chicken	SRR10538025
18V3085F2	*C. jejuni*	46	Chicken	SRR10537836
19N3083F1	*C. jejuni*	46	Chicken	SRR10537616
19N3111F1	*C. jejuni*	46	Pork	SRR10537607
17N3105F1	*C. jejuni*	48	Chicken	SRR10537762
17V3029F1	*C. jejuni*	48	Chicken	SRR10537968
18A3011F1	*C. jejuni*	48	Chicken	SRR10537712
18A3012F1	*C. jejuni*	48	Lamb	SRR10537701
18V3078F1	*C. jejuni*	48	Chicken	SRR10537844
17N3027F1	*C. jejuni*	49	Chicken	SRR10537804
17Q3111F1	*C. jejuni*	49	Chicken	SRR10537463
17Q3127F1	*C. jejuni*	49	Chicken	SRR10538061
17V3012F2	*C. jejuni*	49	Chicken	SRR10537983
17V3033F1	*C. jejuni*	49	Chicken	SRR10537961
17N3042F1	*C. jejuni*	50	Lamb	SRR10537794
17N3053F1	*C. jejuni*	50	Lamb	SRR10537787

17N3062F1	*C. jejuni*	50	Beef	SRR10537783
17N3070F1	*C. jejuni*	50	Lamb	SRR10537777
17N3080F1	*C. jejuni*	50	Beef	SRR10537770
17N3175F1	*C. jejuni*	50	Chicken	SRR10537747
17N3205F1	*C. jejuni*	50	Chicken	SRR10537741
17Q3001F4	*C. jejuni*	50	Beef	SRR10537588
17Q3012F1	*C. jejuni*	50	Lamb	SRR10537576
17Q3014F1	*C. jejuni*	50	Lamb	SRR10537574
17Q3018F1	*C. jejuni*	50	Lamb	SRR10537570
17Q3056F1	*C. jejuni*	50	Chicken	SRR10537529
17Q3062F1	*C. jejuni*	50	Chicken	SRR10537522
17Q3066F1	*C. jejuni*	50	Chicken	SRR10537518
17Q3071F1	*C. jejuni*	50	Chicken	SRR10537513
17Q3074F1	*C. jejuni*	50	Chicken	SRR10537509
17Q3082F1	*C. jejuni*	50	Chicken	SRR10537500
17Q3092F1	*C. jejuni*	50	Lamb	SRR10537483
17Q3093F1	*C. jejuni*	50	Lamb	SRR10537482
17Q3097F1	*C. jejuni*	50	Chicken	SRR10537477
17Q3102F1	*C. jejuni*	50	Chicken	SRR10537472
17Q3108F1	*C. jejuni*	50	Chicken	SRR10537466
17Q3113F1	*C. jejuni*	50	Chicken	SRR10537461
17Q3114F2	*C. jejuni*	50	Chicken	SRR10537460
17Q3136F1	*C. jejuni*	50	Chicken	SRR10538054
18A3015F1	*C. jejuni*	50	Lamb	SRR10537667
18A3021F1	*C. jejuni*	50	Chicken	SRR10537601
18A3025F1	*C. jejuni*	50	Chicken	SRR10537545
18A3047F1	*C. jejuni*	50	Lamb	SRR10538040
18A3048F1	*C. jejuni*	50	Lamb	SRR10538029
18A3050F1	*C. jejuni*	50	Lamb	SRR10538007
18A3064F1	*C. jejuni*	50	Lamb	SRR10537840
18N3002F1	*C. jejuni*	50	Lamb	SRR10537717
18N3013F1	*C. jejuni*	50	Pork	SRR10537708
18N3022F1	*C. jejuni*	50	Lamb	SRR10537703
18N3023F1	*C. jejuni*	50	Lamb	SRR10537702
18N3050F1	*C. jejuni*	50	Chicken	SRR10537683
18N3090F1	*C. jejuni*	50	Pork	SRR10537642
18N3148F1	*C. jejuni*	50	Lamb	SRR10537628
18Q3010F1	*C. jejuni*	50	Pork	SRR10538037
18Q3011F1	*C. jejuni*	50	Pork	SRR10538036
18Q3012F1	*C. jejuni*	50	Chicken	SRR10538035
18Q3020F1	*C. jejuni*	50	Chicken	SRR10538026
18Q3030F1	*C. jejuni*	50	Chicken	SRR10538016
18Q3034F1	*C. jejuni*	50	Chicken	SRR10538012
18V3025F1	*C. jejuni*	50	Chicken	SRR10537908
18V3038F1	*C. jejuni*	50	Chicken	SRR10537893
18V3046F1	*C. jejuni*	50	Chicken	SRR10537883
19N3103F1	*C. jejuni*	50	Chicken	SRR10537614
19N3105F1	*C. jejuni*	50	Beef	SRR10537613
19N3116F1	*C. jejuni*	50	Pork	SRR10537603
17N3055F1	*C. jejuni*	51	Chicken	SRR10537786

17N3072F1	*C. jejuni*	51	Chicken	SRR10537775
17V3028F1	*C. jejuni*	51	Chicken	SRR10537969
18A3007F1	*C. jejuni*	51	Chicken	SRR10537797
18A3007F2	*C. jejuni*	51	Chicken	SRR10537789
18A3054F1	*C. jejuni*	51	Chicken	SRR10537962
18N3094F1	*C. jejuni*	51	Chicken	SRR10537638
19N3191F1	*C. jejuni*	52	Chicken	SRR10537592
17N3031F1	*C. jejuni*	53	Beef	SRR10537800
17Q3004F1	*C. jejuni*	53	Beef	SRR10537585
17Q3016F1	*C. jejuni*	53	Lamb	SRR10537572
17Q3057F1	*C. jejuni*	53	Chicken	SRR10537528
18A3029F1	*C. jejuni*	53	Chicken	SRR10537501
18A3030F1	*C. jejuni*	53	Chicken	SRR10537490
18V3024F1	*C. jejuni*	53	Chicken	SRR10537909
19N3185F1	*C. jejuni*	53	Chicken	SRR10537595
17N3016F1	*C. jejuni*	61	Lamb	SRR10537813
17N3090F1	*C. jejuni*	61	Chicken	SRR10537766
17N3155F1	*C. jejuni*	61	Beef	SRR10537749
17Q3002F1	*C. jejuni*	61	Beef	SRR10537587
17Q3005F1	*C. jejuni*	61	Beef	SRR10537584
17Q3010F1	*C. jejuni*	61	Lamb	SRR10537578
17Q3011F1	*C. jejuni*	61	Lamb	SRR10537577
17Q3013F1	*C. jejuni*	61	Lamb	SRR10537575
17Q3017F1	*C. jejuni*	61	Lamb	SRR10537571
17Q3039F1	*C. jejuni*	61	Chicken	SRR10537546
17Q3090F1	*C. jejuni*	61	Beef	SRR10537484
17Q3132F1	*C. jejuni*	61	Beef	SRR10538058
17Q3135F1	*C. jejuni*	61	Lamb	SRR10538055
18A3016F1	*C. jejuni*	61	Beef	SRR10537656
18A3032F1	*C. jejuni*	61	Lamb	SRR10537479
18A3049F1	*C. jejuni*	61	Beef	SRR10538018
18A3067F1	*C. jejuni*	61	Lamb	SRR10537829
18N3014F1	*C. jejuni*	61	Lamb	SRR10537707
18N3057F1	*C. jejuni*	61	Beef	SRR10537675
18N3064F1	*C. jejuni*	61	Lamb	SRR10537671
18Q3002F1	*C. jejuni*	61	Beef	SRR10538045
18Q3003F1	*C. jejuni*	61	Lamb	SRR10538044
18V3066F2	*C. jejuni*	128	Chicken	SRR10537861
18V3004F1	*C. jejuni*	132	Chicken	SRR10537930
18V3061F1	*C. jejuni*	132	Chicken	SRR10537867
18A3044F1	*C. jejuni*	137	Chicken	SRR10537456
18V3036F2	*C. jejuni*	137	Chicken	SRR10537895
18V3084F1	*C. jejuni*	137	Chicken	SRR10537838
17Q3134F1	*C. jejuni*	190	Lamb	SRR10538056
18N3036F1	*C. jejuni*	190	Lamb	SRR10537688
18Q3005F1	*C. jejuni*	190	Lamb	SRR10538042
18V3050F1	*C. jejuni*	190	Chicken	SRR10537879
19N3110F1	*C. jejuni*	190	Lamb	SRR10537608
17V3035F1	*C. jejuni*	227	Chicken	SRR10537959
18V3043F1	*C. jejuni*	227	Chicken	SRR10537887

17Q3087F1	*C. jejuni*	257	Chicken	SRR10537491
17Q3101F1	*C. jejuni*	257	Chicken	SRR10537473
17Q3121F1	*C. jejuni*	257	Chicken	SRR10538068
18N3031F1	*C. jejuni*	257	Chicken	SRR10537693
18N3037F1	*C. jejuni*	257	Beef	SRR10537687
17N3014F1	*C. jejuni*	449	Chicken	SRR10537815
17N3087F1	*C. jejuni*	449	Chicken	SRR10537768
17N3131F1	*C. jejuni*	449	Chicken	SRR10537758
17Q3048F1	*C. jejuni*	449	Chicken	SRR10537537
17Q3055F1	*C. jejuni*	449	Chicken	SRR10537530
17Q3061F1	*C. jejuni*	449	Chicken	SRR10537524
17Q3080F1	*C. jejuni*	449	Chicken	SRR10537503
17Q3120F1	*C. jejuni*	449	Chicken	SRR10538069
17V3022F1	*C. jejuni*	449	Chicken	SRR10537972
18Q3031F1	*C. jejuni*	449	Chicken	SRR10538015
18Q3037F1	*C. jejuni*	449	Chicken	SRR10538009
18Q3038F1	*C. jejuni*	449	Chicken	SRR10538008
17N3013F1	*C. jejuni*	528	Chicken	SRR10537816
17N3076F1	*C. jejuni*	528	Chicken	SRR10537773
17N3177F1	*C. jejuni*	528	Chicken	SRR10537746
17N3242F1	*C. jejuni*	528	Chicken	SRR10537731
17Q3109F1	*C. jejuni*	528	Chicken	SRR10537465
18N3066F1	*C. jejuni*	528	Chicken	SRR10537669
18V3014F1	*C. jejuni*	528	Chicken	SRR10537921
18V3068F2	*C. jejuni*	528	Chicken	SRR10537858
18V3079F1	*C. jejuni*	528	Chicken	SRR10537843
19N3023F1	*C. jejuni*	528	Chicken	SRR10537621
19N3055F1	*C. jejuni*	528	Beef	SRR10537619
17N3009F1	*C. jejuni*	535	Pork	SRR10537818
17V3052F1	*C. jejuni*	535	Chicken	SRR10537944
18V3054F1	*C. jejuni*	535	Chicken	SRR10537875
18V3062F1	*C. jejuni*	535	Chicken	SRR10537866
18V3063F1	*C. jejuni*	535	Chicken	SRR10537865
18V4087F2	*C. jejuni*	535	Chicken	SRR10537834
17Q3100F1	*C. jejuni*	538	Chicken	SRR10537474
18N3008F1	*C. jejuni*	538	Pork	SRR10537711
18N3009F1	*C. jejuni*	538	Lamb	SRR10537710
18V3028F1	*C. jejuni*	538	Chicken	SRR10537904
18V3048F1	*C. jejuni*	538	Chicken	SRR10537881
17V3042F1	*C. jejuni*	567	Chicken	SRR10537952
18A3028F1	*C. jejuni*	567	Chicken	SRR10537512
18Q3027F1	*C. jejuni*	567	Chicken	SRR10538020
17N3030F1	*C. jejuni*	583	Lamb	SRR10537801
17Q3098F1	*C. jejuni*	583	Chicken	SRR10537476
17V3015F1	*C. jejuni*	583	Chicken	SRR10537980
17V3031F1	*C. jejuni*	583	Chicken	SRR10537966
18Q3015F1	*C. jejuni*	583	Chicken	SRR10538032
18V3047F1	*C. jejuni*	583	Chicken	SRR10537882
17N3061F1	*C. jejuni*	658	Chicken	SRR10537784
17N3073F1	*C. jejuni*	658	Chicken	SRR10537774

18N3076F1	*C. jejuni*	692	Chicken	SRR10537658
18N3073F1	*C. jejuni*	696	Chicken	SRR10537661
17Q3067F1	*C. jejuni*	699	Chicken	SRR10537517
17N3026F1	*C. coli*	825	Chicken	SRR10537805
17N3028F1	*C. coli*	825	Chicken	SRR10537803
17Q3063F1	*C. coli*	825	Chicken	SRR10537521
17Q3085F2	*C. coli*	825	Chicken	SRR10537494
17V3001F1	*C. coli*	825	Chicken	SRR10537995
17V3004F1	*C. coli*	825	Chicken	SRR10537993
17V3005F1	*C. coli*	825	Chicken	SRR10537992
17V3010F1	*C. coli*	825	Chicken	SRR10537987
17V3016F1	*C. coli*	825	Chicken	SRR10537979
17V3017F1	*C. coli*	825	Chicken	SRR10537978
17V3024F1	*C. coli*	825	Chicken	SRR10537970
17V3034F1	*C. coli*	825	Chicken	SRR10537960
17V3036F1	*C. coli*	825	Chicken	SRR10537958
17V3037F1	*C. coli*	825	Chicken	SRR10537957
17V3038F1	*C. coli*	825	Chicken	SRR10537956
17V3041F1	*C. coli*	825	Chicken	SRR10537953
17V3044F1	*C. coli*	825	Chicken	SRR10537950
17V3046F1	*C. coli*	825	Chicken	SRR10537949
17V3047F1	*C. coli*	825	Chicken	SRR10537948
17V3049F1	*C. coli*	825	Chicken	SRR10537946
17V3051F1	*C. coli*	825	Chicken	SRR10537945
17V3056F1	*C. coli*	825	Chicken	SRR10537941
17V3060F2	*C. coli*	825	Chicken	SRR10537934
18N3092F1	*C. coli*	825	Chicken	SRR10537640
18Q3016F1	*C. coli*	825	Chicken	SRR10538031
18Q3017F1	*C. coli*	825	Chicken	SRR10538030
18V3002F1	*C. coli*	825	Chicken	SRR10537932
18V3009F1	*C. coli*	825	Chicken	SRR10537925
18V3015F1	*C. coli*	825	Chicken	SRR10537920
18V3033F1	*C. coli*	825	Chicken	SRR10537899
18V3064F1	*C. coli*	825	Chicken	SRR10537864
18V3081F1	*C. coli*	825	Chicken	SRR10537841
19N3035F1	*C. coli*	825	Chicken	SRR10537620
19N3126F1	*C. coli*	825	Chicken	SRR10537599
17N3001F1	*C. coli*	827	Chicken	SRR10537820
17N3029F1	*C. coli*	827	Lamb	SRR10537802
17N3046F1	*C. coli*	827	Chicken	SRR10537828
17N3048F1	*C. coli*	827	Chicken	SRR10537826
17Q3009F1	*C. coli*	827	Lamb	SRR10537580
17Q3021F1	*C. coli*	827	Lamb	SRR10537565
17Q3028F1	*C. coli*	827	Chicken	SRR10537558
17Q3030F1	*C. coli*	827	Chicken	SRR10537555
17Q3032F1	*C. coli*	827	Chicken	SRR10537553
17Q3043F1	*C. coli*	827	Chicken	SRR10537542
17Q3058F1	*C. coli*	827	Chicken	SRR10537527
17Q3065F1	*C. coli*	827	Chicken	SRR10537519
17Q3115F1	*C. coli*	827	Chicken	SRR10537459

17Q3144F1	*C. coli*	827	Lamb	SRR10538046
17V3006F1	*C. coli*	827	Chicken	SRR10537991
17V3013F1	*C. coli*	827	Chicken	SRR10537982
18A3010F1	*C. coli*	827	Chicken	SRR10537734
18A3010F2	*C. coli*	827	Chicken	SRR10537723
18A3013F1	*C. coli*	827	Chicken	SRR10537690
18A3018F1	*C. coli*	827	Chicken	SRR10537634
18A3019F1	*C. coli*	827	Chicken	SRR10537623
18A3027F1	*C. coli*	827	Chicken	SRR10537523
18N3007F1	*C. coli*	827	Lamb	SRR10537713
18N3012F1	*C. coli*	827	Chicken	SRR10537709
18N3015F1	*C. coli*	827	Pork	SRR10537706
18N3019F1	*C. coli*	827	Pork	SRR10537704
18N3059F1	*C. coli*	827	Lamb	SRR10537674
18N3127F1	*C. coli*	827	Chicken	SRR10537636
18N3135F1	*C. coli*	827	Chicken	SRR10537631
18Q3004F1	*C. coli*	827	Lamb	SRR10538043
18Q3040F1	*C. coli*	827	Chicken	SRR10538005
18V3010F1	*C. coli*	827	Chicken	SRR10537924
18V3013F1	*C. coli*	827	Chicken	SRR10537922
18V3016F1	*C. coli*	827	Chicken	SRR10537919
18V3016F2	*C. coli*	827	Chicken	SRR10537917
18V3017F1	*C. coli*	827	Chicken	SRR10537916
18V3018F1	*C. coli*	827	Chicken	SRR10537915
18V3023F1	*C. coli*	827	Chicken	SRR10537910
18V3026F1	*C. coli*	827	Chicken	SRR10537906
18V3037F1	*C. coli*	827	Chicken	SRR10537894
18V3038F2	*C. coli*	827	Chicken	SRR10537892
18V3039F1	*C. coli*	827	Chicken	SRR10537891
18V3041F1	*C. coli*	827	Chicken	SRR10537889
18V3053F1	*C. coli*	827	Chicken	SRR10537876
18V3056F1	*C. coli*	827	Chicken	SRR10537872
18V3058F1	*C. coli*	827	Chicken	SRR10537870
18V3060F1	*C. coli*	827	Chicken	SRR10537868
18V3067F1	*C. coli*	827	Chicken	SRR10537860
18V3071F2	*C. coli*	827	Chicken	SRR10537854
18V3076F1	*C. coli*	827	Chicken	SRR10537846
18V3077F1	*C. coli*	827	Chicken	SRR10537845
18V3080F1	*C. coli*	827	Chicken	SRR10537842
18V3082F1	*C. coli*	827	Chicken	SRR10537839
18V3086F1	*C. coli*	827	Chicken	SRR10537835
19N3108F1	*C. coli*	827	Beef	SRR10537610
19N3119F1	*C. coli*	827	Chicken	SRR10537602
17N3259F1	*C. coli*	828	Chicken	SRR10537720
18A3004F1	*C. coli*	828	Chicken	SRR10537457
18A3004F2	*C. coli*	828	Chicken	SRR10537963
17V3002F1	*C. coli*	829	Chicken	SRR10537994
17V3014F1	*C. coli*	829	Chicken	SRR10537981
18A3005F1	*C. coli*	829	Chicken	SRR10537852
18A3005F2	*C. coli*	829	Chicken	SRR10537819

18A3059F1	*C. coli*	829	Chicken	SRR10537918
18V3001F1	*C. coli*	829	Chicken	SRR10537933
18V3068F1	*C. coli*	829	Chicken	SRR10537859
17N3044F1	*C. coli*	832	Chicken	SRR10537792
17N3050F1	*C. coli*	832	Pork	SRR10537824
17N3227F1	*C. coli*	832	Chicken	SRR10537735
17N3251F1	*C. coli*	832	Chicken	SRR10537724
17Q3037F1	*C. coli*	832	Chicken	SRR10537548
17Q3038F1	*C. coli*	832	Chicken	SRR10537547
17Q3077F1	*C. coli*	832	Chicken	SRR10537506
17Q3138F1	*C. coli*	832	Chicken	SRR10538052
18A3060F1	*C. coli*	832	Chicken	SRR10537907
18A3060F2	*C. coli*	832	Chicken	SRR10537896
18N3001F1	*C. coli*	832	Chicken	SRR10537718
18N3051F1	*C. coli*	832	Chicken	SRR10537682
18Q3014F1	*C. coli*	832	Chicken	SRR10538033
18Q3023F1	*C. coli*	832	Chicken	SRR10538024
18V3003F1	*C. coli*	832	Chicken	SRR10537931
18V3044F1	*C. coli*	832	Chicken	SRR10537886
19N3195F1	*C. coli*	832	Chicken	SRR10537589
18N3079F1	*C. coli*	854	Pork	SRR10537654
18Q3052F1	*C. coli*	855	Pork	SRR10537997
17N3047F1	*C. coli*	860	Chicken	SRR10537827
17N3067F1	*C. coli*	860	Chicken	SRR10537780
17N3219F1	*C. coli*	860	Chicken	SRR10537737
17Q3031F1	*C. coli*	860	Chicken	SRR10537554
17Q3075F1	*C. coli*	860	Chicken	SRR10537508
17Q3083F2	*C. coli*	860	Chicken	SRR10537498
17Q3112F1	*C. coli*	860	Chicken	SRR10537462
17Q3119F1	*C. coli*	860	Chicken	SRR10538070
18A3002F1	*C. coli*	860	Chicken	SRR10537790
18A3002F2	*C. coli*	860	Chicken	SRR10537679
18A3003F1	*C. coli*	860	Chicken	SRR10537568
18A3006F1	*C. coli*	860	Chicken	SRR10537808
18A3009F1	*C. coli*	860	Chicken	SRR10537756
18A3009F2	*C. coli*	860	Chicken	SRR10537745
18A3022F1	*C. coli*	860	Chicken	SRR10537590
18A3022F2	*C. coli*	860	Chicken	SRR10537579
18N3030F1	*C. coli*	860	Chicken	SRR10537694
18N3074F1	*C. coli*	860	Chicken	SRR10537660
18N3084F1	*C. coli*	860	Chicken	SRR10537649
18N3085F1	*C. coli*	860	Lamb	SRR10537648
18N3086F1	*C. coli*	860	Chicken	SRR10537647
18N3123F1	*C. coli*	860	Chicken	SRR10537637
18N3128F1	*C. coli*	860	Chicken	SRR10537635
18N3129F1	*C. coli*	860	Chicken	SRR10537633
18N3133F1	*C. coli*	860	Chicken	SRR10537632
18N3138F1	*C. coli*	860	Lamb	SRR10537630
19N3022F1	*C. coli*	860	Chicken	SRR10537622
19N3115F1	*C. coli*	860	Chicken	SRR10537604

18V3027F1	*C. coli*	894	Chicken	SRR10537905
18V3030F1	*C. coli*	894	Chicken	SRR10537902
17N3093F1	*C. coli*	966	Chicken	SRR10537763
17N3110F1	*C. coli*	966	Chicken	SRR10537761
17Q3089F1	*C. coli*	966	Chicken	SRR10537486
17Q3089F2	*C. coli*	966	Chicken	SRR10537485
17Q3084F1	*C. jejuni*	991	Chicken	SRR10537497
18V3074F1	*C. jejuni*	991	Chicken	SRR10537848
18Q3032F1	*C. jejuni*	992	Chicken	SRR10538014
18A3056F1	*C. jejuni*	996	Chicken	SRR10537940
17N3207F1	*C. coli*	1016	Pork	SRR10537739
17Q3015F1	*C. coli*	1016	Lamb	SRR10537573
17N3153F1	*C. coli*	1100	Pork	SRR10537751
18N3048F1	*C. coli*	1100	Pork	SRR10537684
18Q3008F1	*C. coli*	1100	Pork	SRR10538039
17Q3081F1	*C. coli*	1173	Chicken	SRR10537502
18V3005F1	*C. coli*	1177	Chicken	SRR10537928
17N3015F1	*C. coli*	1181	Chicken	SRR10537814
17N3049F1	*C. coli*	1181	Chicken	SRR10537825
17N3079F1	*C. coli*	1181	Chicken	SRR10537771
17N3091F1	*C. coli*	1181	Beef	SRR10537765
17N3141F1	*C. coli*	1181	Beef	SRR10537755
17N3143F1	*C. coli*	1181	Chicken	SRR10537754
17N3146F1	*C. coli*	1181	Chicken	SRR10537753
17N3172F1	*C. coli*	1181	Chicken	SRR10537748
17N3180F1	*C. coli*	1181	Chicken	SRR10537744
17N3240F1	*C. coli*	1181	Chicken	SRR10537733
17N3243F1	*C. coli*	1181	Chicken	SRR10537730
17N3246F1	*C. coli*	1181	Chicken	SRR10537728
17N3248F1	*C. coli*	1181	Chicken	SRR10537726
17Q3025F1	*C. coli*	1181	Chicken	SRR10537561
17Q3026F1	*C. coli*	1181	Chicken	SRR10537560
17Q3036F1	*C. coli*	1181	Chicken	SRR10537549
17Q3042F1	*C. coli*	1181	Chicken	SRR10537543
17Q3105F1	*C. coli*	1181	Chicken	SRR10537469
17Q3106F1	*C. coli*	1181	Chicken	SRR10537467
17Q3137F1	*C. coli*	1181	Chicken	SRR10538053
17V3011F1	*C. coli*	1181	Chicken	SRR10537986
17V3012F1	*C. coli*	1181	Chicken	SRR10537984
17V3019F1	*C. coli*	1181	Chicken	SRR10537976
17V3039F1	*C. coli*	1181	Chicken	SRR10537955
17V3048F1	*C. coli*	1181	Chicken	SRR10537947
17V3055F2	*C. coli*	1181	Chicken	SRR10537942
17V3058F1	*C. coli*	1181	Chicken	SRR10537938
17V3059F1	*C. coli*	1181	Chicken	SRR10537936
18A3065F1	*C. coli*	1181	Chicken	SRR10537832
18A3065F2	*C. coli*	1181	Chicken	SRR10537831
18N3055F1	*C. coli*	1181	Chicken	SRR10537677
18N3067F1	*C. coli*	1181	Chicken	SRR10537668
18N3083F1	*C. coli*	1181	Chicken	SRR10537650

18N3087F1	*C. coli*	1181	Chicken	SRR10537646
18N3088F1	*C. coli*	1181	Chicken	SRR10537644
18N3093F1	*C. coli*	1181	Chicken	SRR10537639
18Q3009F1	*C. coli*	1181	Pork	SRR10538038
18V3006F1	*C. coli*	1181	Chicken	SRR10537927
18V3022F1	*C. coli*	1181	Chicken	SRR10537911
18V3035F1	*C. coli*	1181	Chicken	SRR10537897
18V3045F1	*C. coli*	1181	Chicken	SRR10537884
18V3049F1	*C. coli*	1181	Chicken	SRR10537880
18V3057F1	*C. coli*	1181	Chicken	SRR10537871
18V3066F1	*C. coli*	1181	Chicken	SRR10537862
18V3072F1	*C. coli*	1181	Chicken	SRR10537853
18V3073F2	*C. coli*	1181	Chicken	SRR10537849
18V3075F1	*C. coli*	1181	Chicken	SRR10537847
19N3082F1	*C. coli*	1181	Chicken	SRR10537617
19N3099F1	*C. coli*	1181	Chicken	SRR10537615
19N3106F1	*C. coli*	1181	Chicken	SRR10537611
18A3057F1	*C. coli*	1243	Chicken	SRR10537929
18A3062F1	*C. coli*	1243	Chicken	SRR10537874
18A3063F1	*C. coli*	1243	Chicken	SRR10537863
18A3063F2	*C. coli*	1243	Chicken	SRR10537851
18N3082F1	*C. coli*	1243	Chicken	SRR10537651
18N3075F1	*C. coli*	1416	Pork	SRR10537659
17Q3050F1	*C. coli*	1427	Chicken	SRR10537535
17Q3085F1	*C. coli*	1427	Chicken	SRR10537495
18V3031F1	*C. coli*	1427	Chicken	SRR10537901
18N3029F1	*C. coli*	1546	Pork	SRR10537695
17N3022F1	*C. coli*	1563	Chicken	SRR10537809
17Q3023F1	*C. coli*	1766	Chicken	SRR10537563
18V3032F1	*C. jejuni*	1911	Chicken	SRR10537900
17N3258F1	*C. jejuni*	2083	Chicken	SRR10537721
17V3009F1	*C. jejuni*	2083	Chicken	SRR10537988
17V3032F1	*C. jejuni*	2083	Chicken	SRR10537964
18N3005F1	*C. jejuni*	2083	Beef	SRR10537715
18N3070F1	*C. jejuni*	2083	Chicken	SRR10537664
18N3077F1	*C. jejuni*	2083	Chicken	SRR10537657
18N3078F1	*C. jejuni*	2083	Chicken	SRR10537655
18Q3046F1	*C. jejuni*	2083	Pork	SRR10537999
18Q3047F1	*C. jejuni*	2083	Pork	SRR10537998
18V5088F3	*C. jejuni*	2083	Chicken	SRR10537833
18V3042F1	*C. coli*	2179	Chicken	SRR10537888
18Q3043F2	*C. coli*	2733	Pork	SRR10538001
17N3138F1	*C. coli*	3612	Pork	SRR10537757
17N3257F1	*C. coli*	3985	Pork	SRR10537722
17Q3022F1	*C. coli*	3985	Chicken	SRR10537564
17Q3033F1	*C. coli*	3985	Chicken	SRR10537552
17Q3068F1	*C. coli*	3985	Chicken	SRR10537516
17Q3078F1	*C. coli*	3985	Chicken	SRR10537505
17Q3079F1	*C. coli*	3985	Chicken	SRR10537504
17Q3086F2	*C. coli*	3985	Chicken	SRR10537492

17Q3116F1	*C. coli*	3985	Chicken	SRR10537458
17Q3126F1	*C. coli*	3985	Chicken	SRR10538063
18A3046F1	*C. coli*	3985	Chicken	SRR10538051
18Q3026F1	*C. coli*	3985	Chicken	SRR10538021
18Q3028F1	*C. coli*	3985	Chicken	SRR10538019
18Q3033F1	*C. coli*	3985	Chicken	SRR10538013
18Q3036F1	*C. coli*	3985	Chicken	SRR10538010
18N3052F1	*C. coli*	4044	Chicken	SRR10537681
17N3021F1	*C. coli*	4175	Chicken	SRR10537810
17N3068F1	*C. coli*	4175	Chicken	SRR10537779
17N3084F1	*C. coli*	4175	Chicken	SRR10537769
17N3092F1	*C. coli*	4175	Chicken	SRR10537764
17Q3024F1	*C. coli*	4175	Chicken	SRR10537562
17Q3041F1	*C. coli*	4175	Chicken	SRR10537544
17V3020F1	*C. coli*	4175	Chicken	SRR10537975
18N3017F1	*C. coli*	4175	Chicken	SRR10537705
18N3045F1	*C. coli*	4175	Chicken	SRR10537686
18N3089F1	*C. coli*	4175	Lamb	SRR10537643
18Q3024F1	*C. coli*	4175	Chicken	SRR10538023
18V3085F1	*C. coli*	4175	Chicken	SRR10537837
19N3149F1	*C. coli*	4175	Chicken	SRR10537597
17Q3083F1	*C. jejuni*	4896	Chicken	SRR10537499
17Q3088F1	*C. jejuni*	4896	Chicken	SRR10537488
17Q3088F2	*C. jejuni*	4896	Chicken	SRR10537487
17Q3095F1	*C. jejuni*	4896	Chicken	SRR10537480
17Q3139F1	*C. jejuni*	4896	Chicken	SRR10538050
17V3008F1	*C. jejuni*	4896	Chicken	SRR10537989
17V3018F1	*C. jejuni*	4896	Chicken	SRR10537977
17V3057F1	*C. jejuni*	4896	Chicken	SRR10537939
18A3069F1	*C. jejuni*	4896	Chicken	SRR10537822
18Q3035F1	*C. jejuni*	4896	Chicken	SRR10538011
18Q3039F1	*C. jejuni*	4896	Chicken	SRR10538006
18V3011F1	*C. jejuni*	4896	Chicken	SRR10537923
18V3021F1	*C. jejuni*	4896	Chicken	SRR10537912
17N3032F1	*C. jejuni*	5687	Lamb	SRR10537799
17N3034F1	*C. jejuni*	5687	Beef	SRR10537796
17N3035F1	*C. jejuni*	5687	Lamb	SRR10537795
17N3125F1	*C. jejuni*	5687	Chicken	SRR10537759
17Q3006F1	*C. jejuni*	5687	Beef	SRR10537583
17Q3019F1	*C. jejuni*	5687	Lamb	SRR10537569
17Q3020F1	*C. jejuni*	5687	Lamb	SRR10537566
17Q3073F1	*C. coli*	6159	Chicken	SRR10537510
17Q3142F1	*C. jejuni*	6891	Chicken	SRR10538047
17N3010F1	*C. jejuni*	7323	Pork	SRR10537817
17N3057F1	*C. jejuni*	7323	Chicken	SRR10537785
17Q3029F1	*C. jejuni*	7323	Chicken	SRR10537557
17Q3069F1	*C. jejuni*	7323	Chicken	SRR10537515
17Q3086F1	*C. jejuni*	7323	Chicken	SRR10537493
17Q3125F1	*C. jejuni*	7323	Chicken	SRR10538064
17Q3130F1	*C. jejuni*	7323	Chicken	SRR10538059

17Q3140F1	*C. jejuni*	7323	Chicken	SRR10538049
18N3004F1	*C. jejuni*	7323	Chicken	SRR10537716
18N3025F1	*C. jejuni*	7323	Beef	SRR10537699
18Q3041F1	*C. jejuni*	7323	Pork	SRR10538004
18V3069F1	*C. jejuni*	7323	Chicken	SRR10537857
18V3073F1	*C. jejuni*	7323	Chicken	SRR10537850
19N3192F1	*C. jejuni*	7323	Chicken	SRR10537591
17N3151F1	*C. coli*	9419	Chicken	SRR10537752
17N3241F1	*C. coli*	9419	Chicken	SRR10537732
17N3245F1	*C. coli*	9419	Chicken	SRR10537729
17N3247F1	*C. coli*	9419	Chicken	SRR10537727
17Q3003F1	*C. coli*	9419	Beef	SRR10537586
17Q3059F1	*C. coli*	9419	Chicken	SRR10537526
17Q3103F1	*C. coli*	9419	Chicken	SRR10537471
17Q3104F1	*C. coli*	9419	Chicken	SRR10537470
17Q3110F2	*C. coli*	9419	Chicken	SRR10537464
17V3021F1	*C. coli*	9419	Chicken	SRR10537973
18N3028F1	*C. coli*	9419	Chicken	SRR10537696
18N3032F1	*C. coli*	9419	Chicken	SRR10537692
18N3068F1	*C. coli*	9419	Chicken	SRR10537666
18Q3029F1	*C. coli*	9419	Chicken	SRR10538017
19N3014F1	*C. coli*	9419	Chicken	SRR10537626
19N3188F1	*C. coli*	9419	Chicken	SRR10537594
18A3001F1	*C. coli*	9420	Chicken	SRR10537791
18N3072F1	*C. coli*	9420	Chicken	SRR10537662
19N3109F1	*C. coli*	9420	Lamb	SRR10537609
19N3114F1	*C. coli*	9420	Chicken	SRR10537605
19N3152F1	*C. coli*	9420	Chicken	SRR10537596
17N3221F1	*C. jejuni*	9425	Chicken	SRR10537736
17Q3124F1	*C. jejuni*	9425	Chicken	SRR10538065
17V3058F2	*C. jejuni*	9429	Chicken	SRR10537937
18V3020F1	*C. jejuni*	9429	Chicken	SRR10537913
18V3040F1	*C. jejuni*	9429	Chicken	SRR10537890
18V3051F1	*C. jejuni*	9429	Chicken	SRR10537878
18V3055F1	*C. jejuni*	9429	Chicken	SRR10537873
18V3059F1	*C. jejuni*	9429	Chicken	SRR10537869
17Q3064F1	*C. jejuni*	9432	Chicken	SRR10537520
18A3008F1	*C. jejuni*	9432	Chicken	SRR10537778
18A3008F2	*C. jejuni*	9432	Chicken	SRR10537767
18A3020F1	*C. jejuni*	9432	Chicken	SRR10537612
18A3026F1	*C. jejuni*	9432	Chicken	SRR10537534
18A3061F1	*C. jejuni*	9432	Chicken	SRR10537885
18A3070F1	*C. jejuni*	9432	Chicken	SRR10537821
18N3071F1	*C. jejuni*	9432	Lamb	SRR10537663
18N3091F1	*C. jejuni*	9432	Chicken	SRR10537641
18V3052F1	*C. jejuni*	9432	Chicken	SRR10537877
19N3112F1	*C. jejuni*	9432	Chicken	SRR10537606
17N3051F1	*C. coli*	9433	Pork	SRR10537788
17N3260F1	*C. coli*	9433	Pork	SRR10537719
17Q3053F1	*C. coli*	9433	Chicken	SRR10537532

17N3184F1	*C. coli*	9435	Pork	SRR10537742
17N3206F1	*C. coli*	9435	Chicken	SRR10537740
17N3066F1	*C. coli*	9436	Chicken	SRR10537781
17N3119F1	*C. coli*	9436	Chicken	SRR10537760
17Q3027F1	*C. coli*	9436	Chicken	SRR10537559
17Q3034F1	*C. coli*	9436	Chicken	SRR10537551
17Q3096F1	*C. coli*	9436	Chicken	SRR10537478
18A3014F1	*C. coli*	9436	Chicken	SRR10537678
18N3024F1	*C. coli*	9436	Chicken	SRR10537700
18N3147F1	*C. coli*	9436	Chicken	SRR10537629
19N3017F1	*C. coli*	9436	Chicken	SRR10537625
19N3021F1	*C. coli*	9436	Chicken	SRR10537624
19N3057F1	*C. coli*	9436	Chicken	SRR10537618
19N3121F1	*C. coli*	9436	Chicken	SRR10537600
17Q3099F1	*C. coli*	9789	Chicken	SRR10537475
18Q3018F1	*C. coli*	9789	Chicken	SRR10538028
18Q3019F1	*C. coli*	9789	Chicken	SRR10538027
17V3023F1	*C. coli*	9912	Chicken	SRR10537971
17N3020F1	*C. jejuni*	10123	Chicken	SRR10537811
17Q3054F1	*C. jejuni*	10123	Chicken	SRR10537531
17N3023F1	*C. jejuni*	10124	Chicken	SRR10537807
17N3043F1	*C. jejuni*	10125	Lamb	SRR10537793
17N3078F1	*C. jejuni*	10126	Chicken	SRR10537772
17N3250F1	*C. jejuni*	10127	Chicken	SRR10537725
17Q3123F1	*C. jejuni*	10127	Chicken	SRR10538066
18N3062F1	*C. jejuni*	10128	Pork	SRR10537672
17Q3117F1	*C. jejuni*	10129	Chicken	SRR10538072
17Q3118F1	*C. jejuni*	10130	Chicken	SRR10538071
17Q3141F1	*C. jejuni*	10130	Chicken	SRR10538048
18Q3025F1	*C. jejuni*	10130	Chicken	SRR10538022
18A3066F1	*C. jejuni*	10134	Chicken	SRR10537830
19N3143F1	*C. jejuni*	10134	Chicken	SRR10537598
18A3045F1	*C. jejuni*	10136	Chicken	SRR10538062
18A3053F1	*C. jejuni*	10137	Beef	SRR10537974
18Q3013F1	*C. jejuni*	10139	Chicken	SRR10538034
18N3046F1	*C. jejuni*	10140	Pork	SRR10537685
17N3154F1	*C. coli*	10141	Pork	SRR10537750
17N3215F1	*C. coli*	10142	Pork	SRR10537738
17N3071F1	*C. coli*	10144	Chicken	SRR10537776
17N3181F1	*C. coli*	10144	Chicken	SRR10537743
17Q3035F1	*C. coli*	10144	Chicken	SRR10537550
17Q3070F1	*C. coli*	10144	Chicken	SRR10537514
17N3024F1	*C. coli*	10145	Chicken	SRR10537806
17Q3047F1	*C. coli*	10145	Chicken	SRR10537538
17Q3052F1	*C. coli*	10145	Chicken	SRR10537533
17Q3076F1	*C. coli*	10147	Chicken	SRR10537507
17Q3084F2	*C. coli*	10148	Chicken	SRR10537496
17V3007F1	*C. coli*	10150	Chicken	SRR10537990
18A3055F1	*C. coli*	10151	Chicken	SRR10537951
18N3034F1	*C. coli*	10154	Pork	SRR10537689

18N3080F1	*C. coli*	10156	Chicken	SRR10537653
17Q3129F1	*C. coli*	10159	Chicken	SRR10538060
18Q3007F1	*C. coli*	10159	Pork	SRR10538041
18Q3044F1	*C. coli*	10160	Pork	SRR10538000
18V3029F1	*C. coli*	10162	Chicken	SRR10537903
18V3034F1	*C. coli*	10163	Chicken	SRR10537898
17Q3044F1	*C. coli*	10164	Chicken	SRR10537541
17Q3045F1	*C. coli*	10164	Chicken	SRR10537540
17Q3046F1	*C. coli*	10164	Chicken	SRR10537539
17Q3087F2	*C. coli*	10164	Chicken	SRR10537489
17V3031F2	*C. coli*	10166	Chicken	SRR10537965
18A3052F1	*C. coli*	10167	Pork	SRR10537996
18A3052F2	*C. coli*	10167	Pork	SRR10537985
18N3027F1	*C. coli*	10170	Chicken	SRR10537697
18N3061F1	*C. coli*	10171	Pork	SRR10537673
18Q3042F1	*C. coli*	10174	Pork	SRR10538003
19N3190F1	*C. coli*	10176	Pork	SRR10537593
17N3018F1	*C. coli*	-	Chicken	SRR10537812
17V3040F1	*C. coli*	-	Chicken	SRR10537954
18N3006F1	*C. coli*	-	Chicken	SRR10537714
18V3019F1	*C. coli*	-	Chicken	SRR10537914
